# Surgical Apgar Score and Controlling Nutritional Status Score are significant predictors of major complications after cervical spine surgery

**DOI:** 10.1038/s41598-022-10674-2

**Published:** 2022-04-22

**Authors:** Kousei Miura, Masao Koda, Toru Funayama, Hiroshi Takahashi, Hiroshi Noguchi, Kentaro Mataki, Yosuke Shibao, Kosuke Sato, Fumihiko Eto, Mamoru Kono, Tomoyuki Asada, Masashi Yamazaki

**Affiliations:** Department of Orthopaedic Surgery, Faculty of Medicine, University of Tsukuba, 1-1-1 Tennodai, Tsukuba, Ibaraki 305-8575 Japan

**Keywords:** Risk factors, Neurological disorders

## Abstract

Nutritional screening scores, including Controlling Nutritional Status (CONUT) Score and Surgical Apgar Score (SAS), which reflect intraoperative hemodynamics, have been reported to be useful for predicting major postoperative complications in various kinds of surgery. We assessed independent risk factors for major complications after cervical spine surgery using those scoring measurements. We retrospectively reviewed medical records of patients who underwent cervical spine surgery at our institution from 2014 to 2019. Baseline clinical information, including the CONUT Score, and surgical factors, including the SAS, were assessed as risk factors for major postoperative complications. We analyzed 261 patients. Major postoperative complications occurred in 40 cases (15.3%). In the multivariate analysis, SAS (odds ratio [OR], 0.42; *P* < 0.01), CONUT (OR, 1.39; *P* < 0.01), and operative time (OR, 1.42; *P* < 0.01) were significant independent risk factors of major complications. The area under the SAS curve was 0.852 in the receiver operating characteristic curve analysis. Postoperative hospitalization duration was significantly longer in major complications group. Evaluating preoperative nutritional condition and intraoperative hemodynamics with CONUT score and SAS was useful for predicting major postoperative complications of cervical spine surgery. In addition, both scoring measurements are easily calculated, objective evaluations. Perioperative management utilizing those scoring measurements may help prevent them.

## Introduction

In our worldwide aging society, the number of patients with degenerative cervical disorders has been increasing^1^, and because of this, it has been reported that the number of cervical spine surgeries has increased in recent decades^2^. In addition, these numbers are predicted to increase in the coming decades due to the progression of aging in our society^3^. Improvement of perioperative management and surgical techniques can help us to implement relatively highly invasive cervical spine surgeries, such as for cervical deformity, even in patients with advanced age and/or multiple comorbidities. Those improvements are expected to result in satisfactory postoperative functional recovery in those high-risk patients. In contrast, if major complications, such as those related to vital organs, sepsis, and wound healing problems, occur after cervical spine surgery, poor surgical outcomes will result, and increased medical costs will be incurred. Thus, it is obvious that it is crucial to predict the risk of postoperative complications after cervical spine surgery. Previous studies have identified several risk factors for major postoperative complications, such as advanced age, diabetes mellitus (DM), cerebrovascular disease, malignant tumor, many comorbidities, long surgical time, instrumentation surgery, and surgery for ossification of the posterior longitudinal ligament (OPLL), after cervical spine surgery^4–9^. However, further study is needed to provide stronger evidence for these predictive factors in high-risk patients.

It was reported that a preoperative nutritional screening score and the intraoperative Surgical Apgar Score (SAS) were useful for predicting the occurrence of major post-surgical complications in various kinds of surgery, such as abdominal and vascular surgery. Gawande et al. reported the novel SAS system to predict major postoperative complications after digestive and vascular surgery^10^. The components of the SAS are estimated blood loss, lowest mean arterial pressure, and lowest heart rate during surgery, reflecting intraoperative hemodynamics (Table 1). The SAS has been validated in various surgical subspecialities^11,12^. In addition, the Controlling Nutritional Status (CONUT) Score, which is comprised of serum albumin (ALB), total lymphocyte count (TLC), and total cholesterol (TC), is used to evaluate nutritional status (Table 2)^13^. The CONUT Score (range, 0–10) was divided into the following four groups: normal (range, 0–1), light (range, 2–4), moderate (range, 5–8), and severe (9–10). It has been reported that the CONUT Score could be a prognostic factor in patients with several kinds of cancer, including hepatocellular, gastric, and esophageal carcinoma^14–16^. However, few studies have focused on applying those scoring systems to patients undergoing cervical spine surgery. We hypothesized that the SAS and the CONUT Score could predict the occurrence of major postoperative complications after cervical spine surgery. The present study aimed to assess the relationship between those scoring systems and major complications after surgery.

**Table 1:** Definition of the Controlling Nutritional Status (CONUT) Score.

CONUT	Group			
	Normal	Light	Moderate	Severe
Serum Albumin (ALB) (g/dl)	≧ 3.5	3.0–3.4	2.5–2.9	< 2.5
Score	0	2	4	6
Total Lymphocytes (TLC) (/ml)	≧ 1600	1200–1599	800–1199	< 800
Score	0	1	2	3
Total Cholesterol (TC) (mg/dl)	≧ 180	140–179	100–139	< 100
Score	0	1	2	3
Screening Total Score	0–1	2–4	5–8	9–12

The CONUT Score is calculated as the sum of the ALB score, TLC score, and TC score.

**Table 2:** Definition of the Surgical Apgar Score (SAS).

SAS	Score				
	0	1	2	3	4
Estimated blood loss (ml)	> 1000	601–1000	101–600	≦ 100	–
Lowest mean arterial pressure (mmHg)	< 40	40–54	55–69	≧ 70	–
Lowest heart rate (beats/min)	> 85	76–85	66–75	56–65	≦ 55

The SAS is calculated as the sum of the estimated blood loss score, lowest mean arterial pressure score, and lowest heart rate score.

## Material and methods

### Study design

This was a retrospective case–control study based on patient medical records.

### Patient selection

Patients undergoing cervical spine surgery at our institution from 2014 to 2019 were included in this study. The exclusion criteria were as follows: (1) age less than 18 years, (2) minor surgery (e.g., biopsy, debridement, etc.) or planned staged surgery, (3) inadequate laboratory data and intraoperative anesthesia record. This study design was approved by the ethics committee of the University of Tsukuba Hospital. The present study was performed in accordance with the contemporary amendments of the Declaration of Helsinki and within an appropriate ethical framework. All patients signed informed consent before participating in this study.

### Collected data

Baseline clinical information, including age, gender, body mass index (BMI), comorbidities (DM, hypertension, coronary artery disease, anticoagulation therapy, antiplatelet therapy), preoperative hemoglobin, and American Society of Anesthesiologists Physical Status Classification (ASA classification), was collected. Surgical factors consisting of surgical time, surgical approach, use of implants, multisegment surgery (more than five levels including the occipital segment or below T3) were investigated. The SAS was calculated based on estimated blood loss, lowest heart rate, and lowest mean blood pressure during surgery from computerized anesthesia records to evaluate intraoperative hemodynamics (Table 1)^10^. The CONUT Score was measured by laboratory examination of ALB, TLC, and TC, which was performed within three months preoperatively, as the evaluation of preoperative nutritional condition (Table 2)^13^.

### Definition of major complications

The occurrence of the following postoperative major complications within 30 days after surgery was investigated: unplanned intubation for 48 h or longer, bleeding requiring transfusion of > 4 U red blood cells within 72 h after surgery, coronary artery disease, acute renal failure, stroke or cerebral hemorrhage, sepsis, pneumonia, severe delirium, deep venous thrombosis, pulmonary embolism, and wound disruption, as reported previously by Gawande et al.^10^. The length of required hospital stay from the day of surgery to the day of discharge was assessed.

### Statistical analysis

All statistical analyses were performed using JMP (version 14.0.0; SAS Institute Inc, Cary, NC, USA). Correlations between the occurrence of postoperative major complications and baseline clinical factors, surgery, the SAS, and the CONUT Score were analyzed. First, we carried out a univariate analysis using the Chi-square test and the Fisher's exact test for categorical variables, and the Mann–Whitney test for continuous variables. Continuous variables were expressed as mean ± standard deviation (SD). Next, the variables with *P* < 0.1 in univariate analysis were included in the multivariate analyses. Independent risk factors for major postoperative complications were analyzed by using multivariate logistic regression analyses with a stepwise selection among the selected factors. A receiver operating characteristic (ROC) curve analysis determined the cut-off value. *P* values < 0.05 were considered significant.

## Results

### Patient demographics

**Figure 1:**
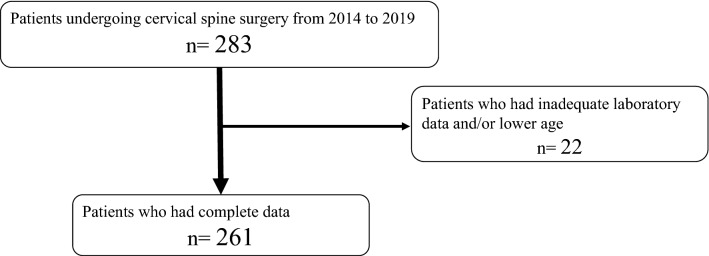
Flow chart for inclusion and exclusion criteria.

Of the 283 patients who satisfied the inclusion criteria, 22 patients were excluded from the present study due to inadequate laboratory data and/or lower age. Finally, 261 patients (172 men and 89 women) who underwent cervical surgery in our institution were analyzed in this study (Fig. 1). The mean age at surgery was 63 ± 13 years (range, 21–87 years), and the mean BMI was 24.1 ± 4.6 kg/m^2^ (range, 14–42 kg/m^2^). The clinical diagnoses were cervical spondylotic myelopathy and/or radiculopathy in 64 cases, ossification of the posterior longitudinal ligament in 64 cases, atlantoaxial subluxation in 29 cases, spinal cord tumor in 18 cases, cervical disc herniation in 17 cases, cervical spondylotic amyotrophy in 12 cases, trauma in 6 cases, cervical deformity in 5 cases, and other in 12 cases.

### Surgical procedure

Anterior, posterior, and combined anterior and posterior surgeries were performed on 49 (18.8%), 194 (74.3%), and 18 (6.9%) patients, respectively. As for the use of implants, 168 patients (64.4%) underwent instrumented fusion surgery with/without decompression.

### SAS and the CONUT Score

The mean SAS was 6.5 ± 1.6. As for the results of the CONUT Score, 167 patients (64.0%) had a normal score, 83 patients (31.8%) had a light score, seven patients (2.7%) had a moderate score, and four patients (1.5%) had a severe score.

### Major complications

Major postoperative complications occurred in 40 cases (15.3%), as shown in Table 3: pneumonia in 14 cases (5.4%), unplanned intubation for 48 h or longer in 9 cases (3.4%), bleeding requiring transfusion of > 4 U red blood cells within 72 h after surgery in 8 cases (3.1%), sepsis in 7 cases (2.7%), severe delirium in 6 cases (2.3%), deep venous thrombosis in 4 cases (1.5%), stroke or cerebral hemorrhage in 3 cases (1.1%), pulmonary embolism in 2 cases (0.8%), and wound disruption in 2 cases (0.8%). Postoperative hospitalization duration was significantly longer in the major complications group than in the no complications group (37 days vs. 20 days, respectively; *p* < 0.01).

**Table 3:** Major Complications.

	*n*	(%)
Overall complications	40	(15.3)
Pneumonia	14	(5.4)
Unplanned intubation for 48 h or longer	9	(3.4)
Bleeding requiring transfusion of > 4 U red blood cells within 72 h after surgery	8	(3.1)
Sepsis	7	(2.7)
Severe delirium	6	(2.3)
Deep venous thrombosis	4	(1.5)
Stroke or cerebral hemorrhage	3	(1.1)
Pulmonary embolism	2	(0.8)
Wound disruption	2	(0.8)

### Analysis for predicting major complications

**Figure 2:**
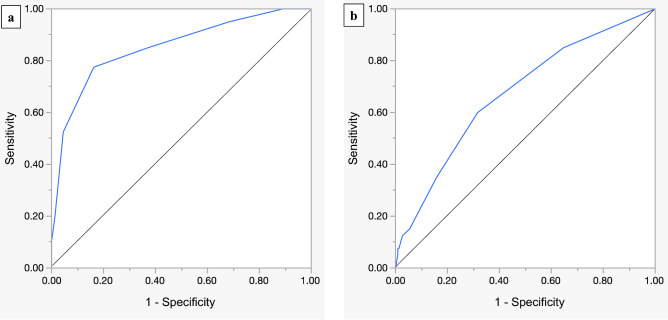
Receiver operating characteristic (ROC) curve analysis for predicting major postoperative complications (**a**) ROC curve of the Surgical Apgar Score (SAS), which shows that the area under the curve (AUC) is 0.852 (**b**) ROC curve of the Controlling Nutritional Status (CONUT) Score, which shows that the AUC is 0.673.

The results of the univariate analyses are shown in Table 4. The mean age in the major complications group was 67 ± 12 years compared to 63 ± 13 years in the no complications group. Among patients with major complications, the mean SAS was 4.6 ± 1.7, and among patients without major complications, the mean SAS was 6.9 ± 1.4. The CONUT Score classification in patients with major complications was as follows: normal in 16 patients (40.0%), light in 19 patients (47.5%), moderate in 3 patients (7.5%), and severe in 2 patients (5.0%). The CONUT Score classification in patients without major complications was as follows: normal in 151 patients (68.3%), light in 64 patients (29.0%), moderate in 4 patients (1.8%), and severe in 2 patients (0.9%). As a result, the univariate analyses showed that significant risk factors of major complications were the following: the presence of DM, higher ASA classification, higher CONUT Score, longer operative time, lower SAS, and combined anterior–posterior surgery and multisegment surgery. In the multivariate analyses, SAS (OR, 0.42; *P* < 0.01), CONUT Score (OR, 1.39; *P* < 0.01), and operative time (OR, 1.42; *P* < 0.01) were estimated as significant independent risk factors of major complications after cervical surgery (Table 5). The ROC curve analysis showed that the optimal cut-off value of the SAS was 5 points, with a sensitivity of 77.5%, a specificity of 83.7%, and an area under the curve (AUC) of 0.852 (Fig. 2a). The ROC curve analysis showed that the optimal cut-off value of the CONUT Score was 2 points, with a sensitivity of 60.0%, a specificity of 68.4%, and an AUC of 0.673 (Fig. 2b).

**Table 4:** Results of Univariate Analyses between the Complications and No-complications Groups (Mean ± SD).

		All cases *n* = 261	Complications *n* = 40	No-Complications *n* = 221	*P*-value
**Background**					
Sex					0.55^†^
	Men	172	28	144	
	Women	89	12	77	
Age (y)		63 ± 13	67 ± 12	63 ± 13	0.078^§^
BMI		24.1 ± 4.6	23.8 ± 5.1	24.2 ± 4.5	0.47^§^
Diabetes mellitus		64	15	49	0.038^†^
Hypertension		108	20	88	0.23^†^
Coronary artery disease		11	3	8	0.38^‡^
Anticoagulant therapy		5	0	5	1^‡^
Antiplatelet therapy		41	10	31	0.079^†^
Preoperative hemoglobin (g/dl)	13.7 ± 1.8	13.0 ± 2.2	13.8 ± 1.7	0.024^§^	
ASA					0.0013^‡^
	1,2	149	13	136	
	3,4	112	27	85	
**Nutrition**					
CONUT Score					0.0047^‡^
	0–1	167	16	151	
	2–4	83	19	64	
	5–8	7	3	4	
	9–12	4	2	2	
**Surgery**					
Operative time		288 ± 138	412 ± 185	265 ± 114	< 0.0001^§^
SAS		6.5 ± 1.6	4.6 ± 1.7	6.9 ± 1.4	< 0.0001^§^
Approach					< 0.0001^†^
	Anterior	49	2	27	
	Posterior	194	29	165	
	AP combined	18	9	9	
Use of implants		168	30	138	0.13^†^
Multisegment surgery (> 5 levels)	39	12	27	0.0037^†^	
**Outcome**					
Postoperative hospitalization	23 ± 14	37 ± 17	20 ± 11	< 0.0001^§^	

^†^Chi square test, ^‡^Fisher exact test, ^§^Mann–Whitney U test.

ASA, American Society of Anesthesiologists physical status; CONUT, Controlling Nutritional Status; SAS, Surgical Apgar Score.

**Table 5:** Results of multivariate analyses for independent predictors of major complications.

	OR	(95% CI)	*P-value*
Age	1.01	(0.98–1.05)	0.41
CONUT	1.39	(1.10–1.77)	0.0061
Operative time	1.42	(1.17–1.72)	0.0001
SAS	0.42	(0.30–0.59)	< 0.0001

CI, confidence interval; OR, odds ratio; CONUT, Controlling Nutritional Status; SAS, Surgical Apgar Score.

## Discussion

These results indicate that the SAS and the CONUT Score are useful independent predictors of major complications after cervical spine surgery. In particular, the SAS showed a relatively higher predictive accuracy compared to the CONUT Score. The SAS is easily calculated from estimated blood loss, heart rate, and mean blood pressure during surgery and can evaluate intraoperative hemodynamics. The CONUT Score, which is also easily calculated from ALB, TLC and TC by preoperative laboratory examination, can reflect preoperative nutritional and inflammatory status. Most importantly, both scoring systems are objective evaluations based on measured values.

However, only a few studies have applied the SAS to spine and neurosurgery. Ou et al. reported that lower scores on the SAS were associated with higher rates of major complications after lumbar fusion surgery for degenerative spine diseases and that the AUC was 0.872 in ROC curve analysis^17^. Ziewacz et al. pointed out that a low SAS could predict 30-day postoperative mortality, complication rates, and extended ICU and hospital stay in neurosurgery^18^. Moreover, according to Urrutia et al., the SAS is a ore useful tool for predicting 30-day postoperative morbidity and mortality in spine surgery compared to general orthopedic surgery^19^. They also reported that the AUC of the SAS was 0.77 in ROC curve analysis for predicting major complications and death after spine surgery^20^. On the other hand, Lau et al.^21^ reported that the SAS was not independently associated with postoperative complications in spinal metastasis, and age and preoperative functional status were stronger predictors. Despite this, they also reported that a low SAS could be an independent predictor of longer hospital stay. Regarding the nutritional condition, several measurements were verified to have an association with postoperative complications after spine surgery. Low prealbumin levels were associated with prolonged length of hospitalization after surgery for cervical myelopathy^22^. An association between the prognostic nutritional index (PNI) and postoperative complications after spine surgery has also been reported. The PNI was an independent risk factor of postoperative delirium after surgery for adult spinal deformity^23^. In addition, a lower preoperative PNI should be considered a risk factor for surgical site infection after spine surgery^24^. However, little has been reported on the association between the CONUT Score and postoperative major complications after cervical spine surgery.

In this study, the occurrence of major postoperative complications after cervical spine surgery significantly extended the length of the required hospital stay. Thus, it is very important to predict the occurrence of major postoperative complications. The present study demonstrated that major complications in high-risk patients could be predicted using the CONUT Score as a preoperative nutritional condition and the SAS as an indicator of intraoperative hemodynamics. Preoperative nutritional intervention may be expected to prevent major postoperative complications after cervical spine surgery in patients with a high CONUT Score. Modifying the surgical strategy to a less invasive surgery should be considered in malnourished patients to avoid an excessive load on intraoperative hemodynamics. More careful postoperative management following cervical spine surgery is thought to be necessary for patients with a low SAS, which indicates poor intraoperative hemodynamics. Perioperative management utilizing the SAS and the CONUT Score holds the promise of preventing major complications after cervical spine surgery.

The present study had several limitations. First, it was a retrospective study based on a review of patient medical records at a single institution with relatively small sample size. Thus, this study might have selection bias and heterogeneity because the decision on whether to perform cervical spine surgery was based on individual cases. Second, we did not evaluate other measurements of nutritional statuses, such as the PNI and prealbumin level. Optimal measurements of nutritional status to predict major postoperative complications are still unclear. Third, the severity of patient comorbidities, which might affect preoperative nutritional status and intraoperative hemodynamics, was not examined. Despite these limitations, the results of this study may be valuable for the perioperative management of patients who undergo cervical spine surgery. Further large-scale prospective studies are needed to confirm these observations.

## Conclusion

This study showed that lower SAS, higher CONUT Score, and longer operative time were significant independent risk factors for major complications after cervical spine surgery in multivariate analysis. The SAS notably revealed a high predictive accuracy similar to previous studies regarding various kinds of surgery. Thus, evaluating the preoperative nutritional status and intraoperative hemodynamics using the SAS and the CONUT Score may be essential for predicting major postoperative complications after cervical spine surgery. In addition, both scoring measurements are easily calculated, objective evaluations. Perioperative management utilizing scoring measurements such as preoperative nutritional intervention, modification of surgical strategy to less invasive surgery for malnourished patients, and more careful postoperative management for patients with poor intraoperative hemodynamics seems possible to prevent major postoperative complications. We suggest that the CONUT Score and the SAS should be evaluated regularly as predictors of major postoperative complications after cervical spine surgery.

## Data Availability

The datasets generated and/or analyzed during the current study are not publicly available due to limitations of ethical approval involving the patient data and anonymity but are available from the corresponding author on reasonable request.

## References

[1] Nouri A (2020). Degenerative cervical myelopathy: A brief review of past perspectives, present developments, and future directions. J. Clin. Med..

[2] Passias PG (2017). Cervical spondylotic myelopathy: National trends in the treatment and perioperative outcomes over 10years. J. Clin. Neurosci..

[3] Neifert SN (2020). Predicting trends in cervical spinal surgery in the United States from 2020 to 2040. World Neurosurg..

[4] Furlan JC, Kalsi-Ryan S, Kailaya-Vasan A, Massicotte EM, Fehlings MG (2011). Functional and clinical outcomes following surgical treatment in patients with cervical spondylotic myelopathy: A prospective study of 81 cases. J Neurosurg Spine.

[5] Cook C (2008). Diabetes and perioperative outcomes following cervical fusion in patients with myelopathy. Spine.

[6] Fehlings MG (2012). Perioperative and delayed complications associated with the surgical treatment of cervical spondylotic myelopathy based on 302 patients from the AOSpine North America Cervical Spondylotic Myelopathy Study. J Neurosurg Spine.

[7] Imajo Y (2017). Surgical and general complications in 2,961 Japanese patients with cervical spondylotic myelopathy: Comparison of different age groups. Spine Surg Relat Res.

[8] Tamai K (2017). Risk factors of cervical surgery related complications in patients older than 80 years. Spine Surg Relat Res.

[9] Tetreault L (2016). Clinical and surgical predictors of complications following surgery for the treatment of cervical spondylotic myelopathy: Results from the multicenter, prospective AOSpine international study of 479 patients. Neurosurgery.

[10] Gawande AA, Kwaan MR, Regenbogen SE, Lipsitz SA, Zinner MJ (2007). An Apgar score for surgery. J. Am. Coll. Surg..

[11] Reynolds PQ, Sanders NW, Schildcrout JS, Mercaldo ND, St Jacques PJ (2011). Expansion of the surgical Apgar score across all surgical subspecialties as a means to predict postoperative mortality. Anesthesiology.

[12] Haynes AB (2011). Surgical outcome measurement for a global patient population: Validation of the Surgical Apgar Score in 8 countries. Surgery.

[13] Ignacio de Ulíbarri J (2005). CONUT: A tool for controlling nutritional status. First validation in a hospital population. Nutr. Hosp..

[14] Wang XB, Chen J, Xiang BD, Wu FX, Li LQ (2019). High CONUT score predicts poor survival and postoperative HBV reactivation in HBV-related hepatocellular carcinoma patients with low HBV-DNA levels. Eur. J. Surg. Oncol..

[15] Kuroda D (2018). Controlling Nutritional Status (CONUT) score is a prognostic marker for gastric cancer patients after curative resection. Gastric Cancer.

[16] Toyokawa T (2016). The pretreatment Controlling Nutritional Status (CONUT) score is an independent prognostic factor in patients with resectable thoracic esophageal squamous cell carcinoma: results from a retrospective study. BMC Cancer.

[17] Ou CY, Hsu SY, Huang JH, Huang YH (2017). Surgical apgar score in patients undergoing lumbar fusion for degenerative spine diseases. Clin. Neurol. Neurosurg..

[18] Ziewacz JE (2013). Validation of the surgical Apgar score in a neurosurgical patient population. J. Neurosurg..

[19] Urrutia J, Valdes M, Zamora T, Canessa V, Briceno J (2012). Can the Surgical Apgar Score predict morbidity and mortality in general orthopaedic surgery?. Int. Orthop..

[20] Urrutia J, Valdes M, Zamora T, Canessa V, Briceno J (2015). An assessment of the Surgical Apgar Score in spine surgery. Spine J..

[21] Lau D, Yee TJ, La Marca F, Patel R, Park P (2017). Utility of the surgical Apgar score for patients who undergo surgery for spinal metastasis. Clin. Spine Surg..

[22] Guan J, Holland CM, Ravindra VM, Bisson EF (2017). Perioperative malnutrition and its relationship to length of stay and complications in patients undergoing surgery for cervical myelopathy. Surg. Neurol. Int..

[23] Oe S (2019). Preoperative age and prognostic nutritional index are useful factors for evaluating postoperative delirium among patients with adult spinal deformity. Spine.

[24] Ushirozako H (2020). Does preoperative prognostic nutrition index predict surgical site infection after spine surgery?. Eur. Spine J..

